# Admixture Mapping Scans Identify a Locus Affecting Retinal Vascular Caliber in Hypertensive African Americans: the Atherosclerosis Risk in Communities (ARIC) Study

**DOI:** 10.1371/journal.pgen.1000908

**Published:** 2010-04-15

**Authors:** Ching-Yu Cheng, David Reich, Tien Y. Wong, Ronald Klein, Barbara E. K. Klein, Nick Patterson, Arti Tandon, Man Li, Eric Boerwinkle, A. Richey Sharrett, W. H. Linda Kao

**Affiliations:** 1 Department of Epidemiology, Johns Hopkins Bloomberg School of Public Health, Baltimore, Maryland, United States of America; 2 Department of Ophthalmology, Taipei Veterans General Hospital, Taipei, Taiwan; 3 Department of Ophthalmology, National Yang Ming University School of Medicine, Taipei, Taiwan; 4 Department of Genetics, Harvard Medical School, Boston, Massachusetts, United States of America; 5 Program in Medical and Population Genetics, Broad Institute of Harvard and Massachusetts Institute of Technology, Boston, Massachusetts, United States of America; 6 Singapore Eye Research Institute and Singapore National Eye Centre, Singapore; 7 Centre for Eye Research Australia, University of Melbourne, Melbourne, Australia; 8 Department of Ophthalmology and Visual Sciences, University of Wisconsin School of Medicine and Public Health, Madison, Wisconsin, United States of America; 9 Human Genetics Center, University of Texas Health Science Center at Houston, Houston, Texas, United States of America; 10 Welch Center for Prevention, Epidemiology, and Clinical Research, Johns Hopkins University, Baltimore, Maryland, United States of America; 11 Department of Medicine, Johns Hopkins University School of Medicine, Baltimore, Maryland, United States of America; University of Chicago, Howard Hughes Medical Institute, United States of America

## Abstract

Retinal vascular caliber provides information about the structure and health of the microvascular system and is associated with cardiovascular and cerebrovascular diseases. Compared to European Americans, African Americans tend to have wider retinal arteriolar and venular caliber, even after controlling for cardiovascular risk factors. This has suggested the hypothesis that differences in genetic background may contribute to racial/ethnic differences in retinal vascular caliber. Using 1,365 ancestry-informative SNPs, we estimated the percentage of African ancestry (PAA) and conducted genome-wide admixture mapping scans in 1,737 African Americans from the Atherosclerosis Risk in Communities (ARIC) study. Central retinal artery equivalent (CRAE) and central retinal vein equivalent (CRVE) representing summary measures of retinal arteriolar and venular caliber, respectively, were measured from retinal photographs. PAA was significantly correlated with CRVE (ρ = 0.071, *P* = 0.003), but not CRAE (ρ = 0.032, *P* = 0.182). Using admixture mapping, we did not detect significant admixture association with either CRAE (genome-wide score = −0.73) or CRVE (genome-wide score = −0.69). An *a priori* subgroup analysis among hypertensive individuals detected a genome-wide significant association of CRVE with greater African ancestry at chromosome 6p21.1 (genome-wide score = 2.31, locus-specific LOD = 5.47). Each additional copy of an African ancestral allele at the 6p21.1 peak was associated with an average increase in CRVE of 6.14 µm in the hypertensives, but had no significant effects in the non-hypertensives (*P* for heterogeneity <0.001). Further mapping in the 6p21.1 region may uncover novel genetic variants affecting retinal vascular caliber and further insights into the interaction between genetic effects of the microvascular system and hypertension.

## Author Summary

Retinal vessels provide a window to microvascular systems elsewhere in the body. The diameter of retinal vessels varies between racial/ethnic groups, being generally wider in African Americans compared to European Americans. To determine whether genetic background may contribute to this observed difference, we scanned the entire genomes of 1,737 African Americans, searching for genomic regions where individuals with either wider retinal venular or narrower retinal arteriolar caliber have a difference from the average percentage of African ancestry. We find that the percentage of African ancestry is positively correlated with retinal venular caliber, particularly in the hypertensive individuals. We detect substantive evidence of association between excess African ancestry and wider retinal venular caliber on chromosome 6p21.1 in the hypertensives, but not in the non-hypertensives. The 6p21.1 region contains genes that are known to be involved in development and modulation and of retinal vessels. Our results suggest that genetic factors may contribute to the observed difference in retinal vascular caliber between African Americans and European Americans. Further fine-mapping studies of the genomic region may identify variants affecting retinal vascular caliber.

## Introduction

Changes in retinal vascular caliber provide unique information regarding the structure and state of the microvasculature in the eye, possibly reflecting pathophysiological processes in the microvascular systems elsewhere in the body. Narrowed retinal arteriolar caliber has been known to be predictive of hypertension [1],[2] and coronary heart disease [3], while wider retinal venular caliber is associated with higher blood pressure [4],[5], impaired fasting glucose, diabetes, dyslipidemia [6]–[8], and risk of coronary heart disease [9]. In particular, because retinal vessels share embryological, anatomical and physiologic characteristics with cerebral vessels [10], wider retinal venular caliber bas been closely linked to both subclinical and clinical cerebrovascular diseases, including lacunar infarction, white matter lesions, clinical stroke [9],[11],[12] and cerebral hypoxia [13].

Retinal vascular caliber has been observed to vary between racial/ethnic groups. In the Multi-Ethnic Study of Atherosclerosis, African Americans and Hispanics had wider retinal arteriolar and venular caliber compared to Whites and Asian Americans, even after controlling for cardiovascular risk factors [8]. In the Atherosclerosis Risk in Communities (ARIC) Study and Cardiovascular Health Study, African Americans had larger retinal arteriolar and venular calibers than European Americans while controlling for age, gender and mean arterial blood pressure [Wong TY, unpublished data, 2009]. The underlying reasons for this racial/ethnic difference are unclear and might be related to systemic, environmental, and measurement factors [14],[15]. However, several lines of evidence provide support for genetic factors also being involved in the regulation of retinal vascular caliber. The heritability of retinal arteriolar and venular caliber was estimated to be 0.48 and 0.54, respectively, in the Beaver Dam Eye Study [16]. Results from two twin studies also showed retinal vascular caliber may be primarily determined by genetic influence with the heritability of 0.57–0.70 for arteriolar caliber and 0.62–0.83 for venular caliber [17],[18]. The observed racial/ethnic differences in retinal vascular caliber could not be fully explained by systemic and environmental factors alone, which prompted our hypothesis that differences in genetic background may partially account for differences in retinal vascular caliber across racial/ethnic populations.

To identify chromosomal regions which may harbor genes that modulate retinal vascular caliber, we utilized admixture mapping, a technique that scans the genomes in recently admixed populations, such as African Americans, for regions which may contain variants that not only differ in frequencies between the two genetically diverse ancestral populations (Europeans and West Africans in this case), but can also partially explain differences in phenotypes between populations [19]–[24]. Since the identification of ancestry-informative markers and the development of appropriate statistical methods for admixture analysis in African Americans [23], admixture mapping and subsequent fine-mapping studies have has been successful in identifying determinant genetic variants for prostate cancer [25],[26], end stage renal disease (ESRD) [27], white blood cell count [28],[29], and the circulating levels of interleukin 6 and interleukin 6 soluble receptor [30]. Differences in retinal vascular caliber between African Americans and European Americans make this an ideal phenotype to study with the admixture mapping approach.

The main hypothesis of the present study was that some alleles affecting retinal vascular caliber are present at higher frequency in Africans than Europeans. We thus conducted a genome-wide admixture mapping scan for retinal arteriolar and venular caliber using approximately 1,365 ancestry informative markers in self-identified blacks from the ARIC study. In addition, hypertension is known as one of the most important risk factors for cardiovascular and cerebrovascular diseases. Previous studies indicated the importance of interactive effects between genes and vascular risk factors, in particular hypertension, on the occurrence of cardiovascular diseases [31]–[33] and cerebrovascular disorders [34]–[36]. It has been suggested that studies investigating the role of genetic components in vascular diseases would be more effective by analyzing interaction of genetic effects with conventional risk factors [35]. Therefore, a secondary purpose of our study was to test the hypothesis that the effect of genetic variants that conferred differences in retinal vascular caliber in African Americans was modified by hypertension.

## Results

Central retinal artery equivalent (CRAE) and central retinal vein equivalent (CRVE) were measured from retinal photographs of study subjects in the ARIC study to represent the average retinal arteriolar and venular caliber, respectively (see Methods). Table 1 shows demographic and phenotypic characteristics of the 1,737 African Americans included in the present study by hypertensive status. Of them, 1,001 (57.9%) had hypertension. The estimated percentage of African ancestry (PAA) in the African-American subjects was 82.4±9.8%.

**Table 1:** Characteristics of the study participants by hypertension status.

Characteristics	With hypertension (N = 1001)	Without hypertension (N = 736)	All (N = 1737)
Age, y	59.0±5.5	57.5±5.1^a^	58.3±5.4
Female, %	66.3	59.8^a^	63.6
Percentage of African ancestry, %	83.0±9.3	81.5±10.7^a^	82.4±9.8
CRAE, µm	162.8±15.6	167.6±15.5^a^	164.8±15.7
CRVE, µm	199.4±16.7	199.9±16.3	199.6±16.5
6-year mean arterial blood pressure^b^, mmHg	98.5±10.2	87.1±7.4^a^	93.7±10.7
Fasting glucose, mg/dL	118.9±47.1	108.4±37.6^a^	114.5±43.6
HDL cholesterol, mg/dL	54.9±18.3	57.1±18.5^a^	55.8±18.4
LDL cholesterol, mg/dL	129.5±36.7	127.6±36.9	128.7±36.8
Triglyceride, mg/dL	119.0±69.8	103.4±59.8^a^	112.4±66.2
Body mass index, kg/m^2^	31.4±6.4	29.0±6.0^a^	30.4±6.3
Diabetes, %	23.4	13.0^a^	19.0
Cigarette smoking, %			
Current	18.9	22.1	20.3
Former	32.7	32.6	32.6
Never	47.4	45.1	46.4
Alcohol consumption, %			
Current	29.0	35.9^a^	31.9
Former	31.0	28.0	29.7
Never	39.0	36.0	37.7

CRAE, central retinal artery equivalent; CRVE, central retinal vein equivalent.

Data are mean ± SD, except for sex, diabetes, cigarette smoking and alcohol consumption, which are presented as percentage, %.

**a** *P*<0.05 for difference between participants with and without hypertension.

**b** Mean arterial pressure, defined as 2/3 diastolic plus 1/3 systolic blood pressure, across the three AIRC visits was averaged to obtain the 6-year mean arterial pressure.

### Correlations of retinal vascular caliber and genetic ancestry

**Figure 1:**
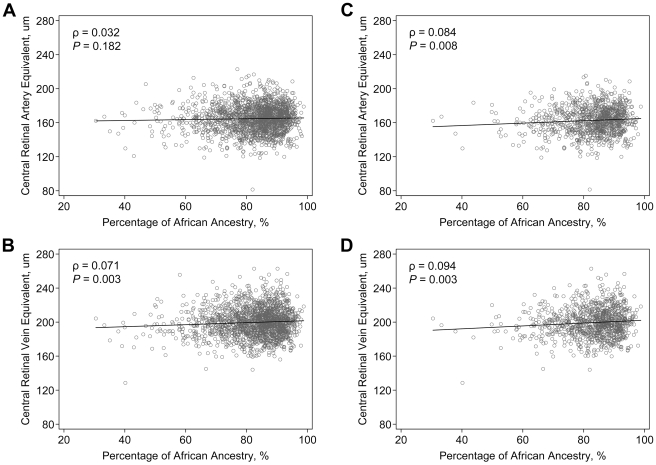
Correlation of retinal vascular calibers and the estimated percentage of African ancestry in the ARIC African Americans. Data are scatter-plotted for (A) central retinal artery equivalent in all individuals, (B) central retinal vein equivalent in all individuals, (C) central retinal artery equivalent in hypertensive individuals, and (D) central retinal vein equivalent in hypertensive individuals. The solid black line in the figure is a linear regression line.

The correlations between retinal vascular caliber and PAA are shown on Figure 1. CRVE was significantly correlated with PAA (correlation coefficient, ρ = 0.071, *P* = 0.003), while the correlation between PAA and CRAE was weaker and not statistically significant (ρ = 0.032, *P* = 0.182). To assess the potential interaction between genetic effects and hypertension, we examined the correlations limited to hypertensive individuals as an *a priori* subgroup analysis. We found that among hypertensive individuals, the correlations with PAA became stronger for both CRAE (ρ = 0.084, *P* = 0.008) and CRVE (ρ = 0.094, *P* = 0.003).

### Initial admixture mapping scans

We carried out genome-wide admixture mapping scans for both CRAE and CRVE using case-only and case-control approaches, using up to 1,365 ancestry-informative SNP markers. Cases (N = 261) and controls (N = 260) for CRAE were defined as the extreme 15% of samples with narrowest caliber and the 15% with widest caliber, respectively, after adjustment for age, sex, study site, 6-year mean blood pressure, and fasting glucose level. For CRVE, the extreme 15% of samples with widest caliber was defined as cases (N = 260), while the extreme 15% with narrowest caliber as controls (N = 261). The mean CRAE was 46.2 µm (±15.3) narrower in cases compared to controls. On the other hand, the mean CRVE was 51.0 µm (±16.1) wider in cases compared to controls. Using 18 pre-specified European ancestry risk models, we did not detect significant admixture association with either CRAE or CRVE (Table S1). The genome-wide score, derived by averaging the evidence of association across all loci examined in the genome, was −0.73 for CRAE and −0.69 for CRVE (Table 2), which did not meet the thresholds of >2 for genome-wide significance [37].

**Table 2:** Summary of locus-specific scores from the admixture scans of retinal vascular caliber.

Samples and traits^a^	No. of SNPs	No. of samples	No. of cases/controls^b^	Genome-wide score	Locus-specific LOD at Chr. 6 peak
All African Americans					
CRAE	1356	1737	261/260	−0.73	0.41
CRVE	1365	1737	260/261	−0.69	1.45
Hypertensive African Americans					
CRAE	1350	1001	151/150	0.18	2.04
CRVE	1359	1001	150/151	2.31^c^	5.47^d^
Non-hypertensive African Americans					
CRAE	1356	736	110/109	−0.40	0.42
CRVE	1357	736	109/110	−0.21	−0.51

CRAE, central retinal artery equivalent; CRVE, central retinal vein equivalent.

**a** Both CRAE and CRVE were adjusted for age, sex, study site, 6-year mean arterial pressure and fasting glucose level.

**b** For CRAE, cases were defined as the 15% with the lowest covariate-adjusted values, and controls were the 15% with the highest covariate-adjusted values. For CRVE, cases were defined as the 15% with the highest covariate-adjusted values, and controls were the 15% with the lowest covariate-adjusted values.

**c** Genome-wide scores >2 are formally significant.

**d** Locus-specific LOD scores >5 are formally significant.

### Significant admixture-generated signal for retinal venular caliber in the hypertensive group

**Figure 2:**
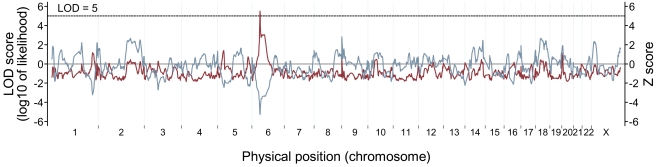
Genome-wide admixture mapping scans for loci affecting retinal venular caliber in hypertensive individuals. For case-only analysis (LOD score, red line), the genome-wide score was 2.31, reaching the threshold of >2 for significance. The highest locus-specific LOD of 5.47 was detected at 6p21.1, and this score again met the threshold of >5 for significance. The admixture-generated signal was further supported by case-control analysis (Z score, blue gray line). At the 6p21.1 peak, the cases had highly significantly lower percentage of European ancestry than the controls (case-control Z score** = **−5.26, *P* **= **1.44×10^−7^).

To examine whether hypertension modified the effects of genetic ancestry on retinal vascular caliber, we performed admixture mapping scans by hypertension status. Cases and controls were defined as the extreme 15% (after adjustment for the covariates as described above) in the hypertensive subset and included about 150 subjects in each group. On average, CRAE was 46.5 µm (±15.5) narrower and CRVE was 51.6 µm (±16.7) wider in the cases, compared to the controls. We found genome-wide significant evidence of associations with CRVE in the hypertensive subset (Table 2). The genome-wide score in the case-only analysis was 2.31, which meets our threshold of >2 for significance. The strongest admixture association for CRVE was observed at 6p21.1 (42.5 Mb on chromosome 6 in build 35 of the human genome reference sequence; Figure 2), with the peak locus-specific LOD of 5.47, again reaching our priori defined thresholds of >5 for significance [37]. To further correct for the multiple hypothesis testing in two subgroups, we divided our test statistic, which is the likelihood ratio that compares the model under a risk model to the null model by 2. The likelihood ratio was 10^5.47^ = 295120.9. The likelihood ratio after corrected for two hypothesis testing was 295120.9/2** = **147560.5, corresponding to a LOD score of 5.17, which still exceeds the threshold of >5 for significance. No other locus exceeded a LOD score of 5.

To evaluate the stability of our results, we carried out a longer analysis with 10-times more iterations in our Markov Chain Monte Carlo run. We obtained a similar strength of signal with a genome-wide score of 2.28 and a peak locus-specific LOD of 5.43 at the same location. The risk model with the strongest score corresponded to a risk of 0.5 due to one copy of an European ancestral allele with the inverse risk for carrying zero copies (see Methods for the set of risk models). Further refining the risk models, we obtain a genome-wide score of 3.66 and a locus-specific LOD of 6.85 in this region.

The admixture-generated signal for CRVE in the hypertensive subset was further supported by the case-control analysis. At the 6p21.1 peak, the cases had a highly statistically significant increase in African ancestry compared to controls (case-control Z score = −5.26, *P* = 1.44×10^−7^; Figure 2). The association was nominally genome-wide significant (*P* = 2.88×10^−4^) after conservatively correcting for multiple hypothesis testing (by multiplying by 2,000 because we tested 1,000 independent chromosomal chunks in two subgroups).

We did not find any significant associations with CRAE in the hypertensive subset (genome-wide score = 0.18). The highest locus-specific LOD was 2.37, arising from chromosome 5, followed by the second highest LOD of 2.04 on chromosome 6 (Figure S1). Admixture scans were also carried out in the non-hypertensive subset, but we did not observe any evidence of association with either CRAE (genome-wide score = −0.40) or CRVE (genome-wide score = −0.21; Table 2).

### Evidence of association between admixture-generated signals and continuous CRVE and interaction with hypertension

To further assess the robustness of the admixture-generated signal, we extracted the local estimate of African ancestry at the 6p21.1peak, and tested for association with continuous CRVE by hypertension status. This enabled us to increase power by including all samples in a quantitative analysis. We carried out a series of linear regression analysis, with the normal-quantile transformed CRVE (after adjusted for covariates as described above) as a dependent variable and the local ancestry as an independent variable. As shown in Table 3, local African ancestry alone was strongly associated with the transformed CRVE in the hypertensive subset (*P* = 2.9×10^−8^; Model 1). To assess whether continuous CRVE was associated with local ancestry at 6p21.1 due to their associations with global ancestry (i.e., PAA), we modeled the transformed CRVE as a function of local, global and regional ancestry. We found that the residual association of local ancestry with the transformed CRVE after adjustment for both global and regional ancestry remained significant (*P* = 3.9×10^−6^), indicating that there may be a gene in the region of 6p21.1 that is associated with CRVE above and beyond the fact that variants in this locus are highly differentiated between ancestral populations and thus correlated with global ancestry. The association was nominally genome-wide significant (*P* = 7.8×10^−3^) after correcting for multiple hypotheses tested (by multiplying by 2,000). The results were similar when CRVE was additionally adjusted for other covariates (Model 2), including high-density lipoprotein (HDL) cholesterol, low-density lipoprotein (LDL) cholesterol and plasma triglyceride levels, body mass index (BMI), smoking, and alcohol consumption. We estimated that each additional copy of an African ancestral allele at the 6p21.1 peak was associated with a CRVE increase of 0.37 Z-score units on average (equivalent to ∼6.14 µm).

**Table 3:** Linear regression analysis of CRVE on local African ancestry on the chromosome 6 peak by hypertension status.

		Model 1^a^			Model 2^b^	
African ancestry and hypertension status	Reg. Coef.^c^	(95% CI)	*P* value	Reg. Coef.^c^	(95% CI)	*P* value
Hypertensive African Americans (n = 1001)						
Local ancestry only	0.36	(0.23, 0.48)	2.9×10^−8^	0.35	(0.22, 0.48)	7.5×10^−8^
Local ancestry, global ancestry as a covariate	0.34	(0.21, 0.48)	7.9×10^−7^	0.34	(0.20, 0.48)	1.6×10^−6^
Local ancestry, global ancestry and regional ancestry covariates	0.38	(0.22, 0.55)	3.9×10^−6^	0.37	(0.21, 0.54)	1.1×10^−5^
Non-hypertensive African Americans (n = 736)						
Local ancestry only	−0.09	(−0.24, 0.06)	0.238	−0.08	(−0.23, 0.07)	0.289
Local ancestry, global ancestry as a covariate	−0.16	(−0.32, 0.01)	0.062	−0.18	(−0.34, −0.01)	0.036
Local ancestry, global ancestry and regional ancestry covariates	−0.12	(−0.32, 0.07)	0.217	−0.14	(−0.33, 0.06)	0.170
Heterogeneity between groups^d^		*P* = 9.2×10^−5^			*P* = 9.5×10^−5^	

CRVE, central retinal vein equivalent; Reg. Coef., regression coefficient of the local ancestry; CI, confidence interval.

**a** CRVE was adjusted for age, sex and study site, 6-year mean arterial pressure and fasting glucose level, and then normal-quantile transformed.

**b** In addition to the variables included in Model 1, CRVE was further adjusted for HDL cholesterol level, LDL cholesterol level, plasma triglyceride level, body mass index, smoking, and alcohol consumption, and then normal-quantile transformed.

**c** The change in Z score for each additional copy of the local ancestry allele.

**d** By Z test for difference between the two the regression coefficients from the hypertensive and non-hypertensive groups in the models with both global and regional ancestry as covariates.

In contrast, in the non-hypertensive subset, the local ancestry effect at the 6p21.1 peak on CRVE was weak and did not reach significance (Table 3). The effect of local ancestry showed significant heterogeneity (*P*<0.001) between the hypertensive and non-hypertensive groups, which was in line with the results of the above dichotomous admixture scans.

### 95% credible interval for the CRVE loci

Given the significant statistical evidence for peak at 6p21.1, we constructed 95% credible interval (CI) for the loci identified. The 95% CI spanned from 40.8 to 43.9 Mb on build 35 of the human genome reference sequence (Figure S2). The locus-specific LOD score and the association of the CRVE to local ancestry for the SNPs located near and within the 95% CI are presented in Table S2.

## Discussion

We used admixture mapping to search for genomic regions that may account for inter-individual variations in retinal vascular caliber. We found evidence for association with retinal venular caliber at 6p21.1 in hypertensive African Americans and observed concordant results when venular caliber was examined as a continuous variable, with higher levels of African ancestry being significantly associated with wider retinal venular caliber. The significant evidence of association with the local ancestry at the 6p21.1 peak was above and beyond the contribution of both global and regional ancestry. Methodologically, these results are interesting in that subset analysis was required in order to detect the association. We note that subsets analysis has previously been very successful in admixture mapping. For prostate cancer, the chromosome 8q24 locus was not detected until the analysis was limited to a subset of individuals with a younger age at diagnosis [25]. For ESRD, the admixture signal to be much stronger among non-diabetic ESRD (mainly hypertensive ESRD) only, compared to diabetic ESRD [27]. Subsequent fine mapping identified genetic variants strongly associated with both prostate cancer [26] and non-diabetic ESRD [27]. We hope to follow up the present analysis with successful fine-mapping as well.

Our findings in persons with hypertension in the subgroup analysis imply that genes associated with hypertension may have exerted their effects on retinal vascular caliber. While we cannot exclude the possibility of a chance finding, we were able to show consistent results in the local ancestry analysis, which included the total population. Furthermore, from a physiological perspective, our finding that there is a genetic association with retinal venular caliber specifically in people with hypertension is sensible. Hypertension is known to have profound effects on the retinal microcirculation [38]–[40], and may induce gene expression relevant to the modulation of retinal vessels (see further discussion below) [41]. Our findings are also in line with previous studies in other vascular diseases that indicated hypertension exaggerates the effects from genetic factors [31]–[36].

In hypertensive persons, we detected significant genetic association for retinal venular caliber, but not arteriolar caliber. Because retinal arterioles and venules likely possess distinct genetic determinants [42], there may be no common genetic variants with a strong effect accounting for differences in retinal arteriolar caliber between European and African populations. Moreover, a diminished capability of retinal arterioles to remodel because of progressive sclerosis and rigidity of arteriolar vessel walls with age [43], may have precluded a degree of change in arteriolar caliber equal to that observed in venular caliber.

To our knowledge, there have been only two prior studies examining the genetic basis of retinal vascular caliber with a genome-wide approach, and both of them used linkage analysis [18],[45]. By genotyping 385 microsatellite markers in the Beaver Dam Eye Study, Xing et al. [45] found several loci for CRAE and CRVE, with the most significant loci at 3q28 (empirical *P* = 1.2×10^−4^) and 8q21 (empirical *P* = 2.9×10^−3^), respectively. A recent linkage analysis in the Australian Twins Eye Study identified 8p23.1 (LOD = 2.24) and 2p14 (LOD = 2.69) as suggestive loci for CRAE and CRVE, respectively [18]. Although findings of the two linkage analyses were not replicated in our study, possibly due to differences in study design and populations, all three studies provide lines of evidence that structural changes in the microvasculature of retina may have genetic determinants.

One major concern of the present study is a potential measurement error on retinal vascular caliber itself, which has been suggested to account for some of the observed racial/ethnic difference in retinal caliber [15]. A recent report suggested that retinal pigmentation could be a source of error in the computer-assisted methods to measure retinal vascular caliber from photographs [15]. The study reported that arteriolar and venular calibers were significantly wider in East Asian children than their white counterparts. However, when the analysis was confined to children with dark brown iris (a surrogate of retinal pigmentation) only, the differences between racial groups were less pronounced. Nevertheless, measurement errors are less likely to bias results in the present study for the following reasons. First, in the ARIC study, the retinal vessel edge was not detected based on computer-generated pixel density curve, but located manually by graders [46]. We believe the manual grading of retinal vascular caliber would be less prone to bias than computerized grading schemes, yet this remains to be proved. Second, genetic ancestry was shown to be significantly correlated with human pigmentation [47]. Although we did not measure skin or retinal pigmentation in the ARIC study, the inclusion of global ancestry as a covariate in our local ancestry analysis (Table 3) may thus provide an alternative way to adjust for the differences in retinal pigmentation.

The 95% CI for the 6p21.1 locus, a ∼3.1 Mb region, contains genes that may have biological relevance to the development and modulation and of retinal vessels (Figure S2). One such gene is the vascular endothelial growth factor (*VEGF*) gene. VEGF is an endothelial-cell selective mitogen intimately associated with vasculogenesis, angiogenesis and vascular permeability. In the retina, VEGF plays a crucial role in the induction of retinal vasculature and its expression is regulated by hypoxia during embryonic development [48]. Moreover, animal experiments showed that VEGF acts as a survival factor for newly formed retinal vessels [49], and it continues to be produced by retinal astrocytes and pericytes in the vicinity of retinal vessels in adults [50]. Interestingly, mechanical stretch on retinal vessel endothelium induced by systemic hypertension could increase the expression of VEGF and its receptor [41]. Further mapping work is needed to determine whether variants in the *VEGF* gene indeed contribute to variations in retinal vascular caliber. If proven, this may help explain why we detected the association at 6p21.1 only in hypertensive persons.

In addition to *VEGF*, there are two other genes in the 95% CI that may potentially be associated with retinal phenotype: peripherin 2 (*PRPH2/RDS*) and guanylate cyclase activator 1A (*GUCA1A*), both of which are also expressed in retina [51],[52]. GUCA1A plays a role in the recovery of retinal photoreceptors from photobleaching [52]. PRPH2/RDS is mainly located in the outer segment of both rod and cone, and defects in this gene are associated with retinal degenerations [51]. *VEGF* appears to be the strongest candidate gene based on its known function. However, it does not exclude the possibility that the admixture-generated signal is due to other genes.

The human leukocyte antigen (HLA) loci, located on chromosome 6p21.3 and about 10 Mb away from the 95% CI, are gene-rich and highly polymorphic [53]. The HLA region has been shown to play an important role in multiple disease susceptibility, particularly in autoimmune and infectious diseases [54]. HLA alleles are strongly associated with many neighboring SNPs, sometimes located at a considerable distance from the HLA allele with the linkage disequilibrium (LD) extending several Mb [55]. It remains to be determined whether the HLA alleles and the alleles in the 95% CI are in LD and may thus be associated with retinal venular caliber.

In summary, using a genome-wide admixture mapping scan in 1,737 African Americans, we detected a risk locus influencing retinal vascular caliber in hypertensive individuals at 6p21.1, where the association between local ancestry and retinal venular caliber was strong, suggesting the presence of a genetic effect beyond the effects of global ancestry. Follow-up fine mapping or haplotype tagging across the peak will be necessary to determine whether this region harbors genetic variations that may interact with hypertension to modulate retinal venular caliber. Understanding the genetic basis of retinal vascular caliber may provide novel insight into the development and remodeling of the microvasculature in the brain and elsewhere in the body.

## Methods

### Ethics statement

This study was conducted according to the principles expressed in the Declaration of Helsinki. All sample collections were carried out according to institutionally approved protocols for study of human subjects and written informed consent was obtained from all subjects.

### Study populations

Subjects of the present study were from the 2,997 African-American participants of the ARIC study at the third examination. The ARIC study is a prospective epidemiologic study that examines clinical and subclinical atherosclerotic disease in a cohort of 15,792 persons (including 4,266 African-Americans), aged 45 to 64 years at their baseline examination. Participants were selected by probability sampling from four U.S. communities: Forsyth County, NC (12% African American); Jackson, MS (100% African American); the northwest suburbs of Minneapolis, MN (<1% African American); and Washington County, MD (<1% African American). The sampling procedure and methods used in ARIC have been described in detail elsewhere [56]. Participants self-reported their ethnicity. The baseline examination (visit 1) took place from 1987 to 1989, a second examination (visit 2) from 1990 to 1992, and a third examination (visit 3) from 1993 to 1995. Retinal vascular calibers were measured at visit 3. Data from visit 3 were used for the present analysis.

The final sample for the present analysis included 1,737 African Americans after excluding the following samples (N = 1,267): *1)* African-American subjects who lived in Minneapolis, MN, or Washington County, MD, or *2)* did not consent to genetic studies or did not have DNA samples available, *3)* samples that were not genotyped successfully or that failed to pass quality control (see “Elimination of problematic samples”), *4)* subjects who had no retinal photographs, ungradable photographs or retinal vascular occlusions, and *5*) subjects who had missing data on the covariates used in the main admixture mapping scans (see “Admixture mapping”).

### Measurement of retinal vascular caliber

The retinal photography procedure and its assessment have been described in detail elsewhere [46]. Briefly, a 45-degree retinal photograph of one randomly selected eye of each participant was taken at visit 3 following 5 minutes of dark adaptation. This photograph was centered on the region of the optic disc and the macula and was taken using an autofocus camera.

Trained graders who were masked to participant measured the calibers of all arterioles and venules coursing through a specified area surrounding the optic disc according to a standardized protocol [46]. Individual vessel measurements were combined into summary indices: CRAE and CRVE. These indices represents average retinal arteriolar and venular caliber of the eye after taking into account the branching patterns. These measurements of retinal vascular calibers are reliable, with generally high intragrader and intergrader reliability coefficients (0.84 and 0.79, respectively) [46],[57].

### Assessment of systemic factors

Current blood pressure was defined as measurements at the time of retinal photography at visit 3, and 3- and 6- year past blood pressure was defined as measurement taken at visit 2 and visit 1, respectively. Mean arterial pressure was defined as two thirds of diastolic plus one third of systolic blood pressure. Mean arterial pressure across the three visits was averaged to obtain the 6-year mean arterial pressure. BMI was calculated as weight (in kg)/height (in meters) squared. Blood collection and processing followed a standard protocol [58]. Fasting glucose was assessed by a modified hexokinase/glucose-6-phosphate dehydrogenase procedure. Total plasma cholesterol and triglyceride were measured using enzymatic methods [59]. LDL cholesterol was calculated [60], and HDL cholesterol was measured after dextran-magnesium precipitation of non-HDL lipoproteins [58]. Cigarette smoking and alcohol consumption were ascertained from a questionnaire interview. Hypertension was defined as current systolic blood pressure ≥140 mm Hg, diastolic pressure ≥90 mm Hg, or self-reported use of medications for high blood pressure during the 2 weeks preceding the clinic examination at visit 3.

### Genotyping for the ancestry informative markers

We genotyped a total of 1,536 SNPs included in the phase 3 admixture panel [28,61]. This panel was constructed by using the panel of ancestry informative markers previously published by Smith et al. (phase 1 panel) [24], improving this panel by mining new ancestry informative markers from the data sets of Hinds et al. [62] and the Phase 2 International Haplotype Map [63], and then validating them to confirm that they were indeed ancestry informative. Genotyping was performed by the Center for Inherited Disease Research (CIDR, Johns Hopkins University, Baltimore), using the Illumina BeadLab platform [64]. The ARIC study has a rigorous quality control program, including blind duplicates. Many genotypes in duplicates were obtained using the Illumina BeadLab technologies in ARIC African-American participants, and CEPH and Yoruban samples. The mismatch rate among duplicate genotypes was 0.1%.

### Estimates of allele frequencies in ancestral populations

We used previously published genotyping data to estimate the frequency of each SNP in West Africans and Europeans [24],[25],[64]. We used only those SNPs for which we were able to obtain data from both West African (Yoruba) and European American (CEU) populations from the International Haplotype Map. For SNPs in the phase 1 panel, we also added additional genotyping data from African and European samples, which was the same as the data collected in Smith et al. 2004 [24].

### Elimination of problematic samples

After genotyping, samples were eliminated based on the following criteria: *1)* samples with low (<94%) call rate, *2)* samples showing gender mismatch between self-reported data and genetically estimated gender based on 50 markers on the X chromosome, and *3)* duplicate samples (defined as >75% match in the genotypes between two samples. Moreover, we used built-in data checking programs in the ANCESTRYMAP [23] software to exclude samples with an apparent excess or deficiency of heterozygous genotypes compared with the expectation from the individuals' global ancestry. An apparent excess of heterozygous genotypes (defined as the Z-score >10) usually indicates the individuals have parents with divergent ancestries (for example, one parent who is entirely of European ancestry) and such individuals nearly always have estimated European ancestry close to 0.5 [23].

### SNP quality filters

To decrease the likelihood of false-positives in our admixture scans, we applied a series of filters that had the goal of detecting and removing any SNPs with problematic genotyping, as described previously [24,30,61,66]. First, SNPs were dropped if there were atypical clustering patterns, ill-defined clusters, or relatively low genotyping success rate (95%). This left us with 1,416 SNPs (all with genotyping call rate >97%). We then applied three previously described filters to further eliminate SNPs from the analysis [66]. *1)* We eliminated SNPs (N = 15) if they did not meet the requirement for Hardy-Weinberg equilibrium (*P*>0.01) in both ancestral West African and European populations. *2)* We applied a “freqcheck” filter that examined whether the observed frequency of a SNP in African Americans was statistically consistent with being a mixture of the frequencies observed in the West Africans and European American samples that we used to represent the ancestral populations [23]. *3)* Lastly, we applied a “ldcheck” filter that for each sample, iteratively eliminated SNPs that were less informative (in terms of the information content about ancestry) until none were within 200 Kbs of each other or in detectable LD with each other in the ancestral West African or European populations [23]. After imposing these requirements, 1,365 SNPs were left for analysis.

### Estimating genome-wide ancestry genetic ancestry

Using the ANCESTRYMAP software [23], we estimated a global ancestry for each individual, as indicated by PAA. ANCESTRYMAP uses a Markov Chain Monte Carlo approach to account for uncertainty in the unknown parameters (including SNP allele frequencies in the West African and European ancestral populations, the number of generations since mixture, and the average proportion of ancestry inherited from ancestral populations) that emerge from the Hidden Markov Model analysis. All Markov Chain Monte Carlo runs used 100 burn-in and 200 follow-on iterations, as recommended [23], except for one longer run of 1,000 burn-in and 2,000 follow-on iterations, which we used to check the stability of our results.

### Admixture mapping

We used the ANCESTRYMAP software [23] to search for genomic regions associated with an increased percentage of either European or African ancestry. The main dichotomous admixture scans used the values of retinal vascular caliber adjusted using the following covariates: age, sex, study sites (Forsyth County or Jackson), 6-year mean arterial blood pressure and fasting glucose level, because the latter two systemic factors were both significantly correlated with PAA (both *P*<0.01) and known to be associated with retinal vascular calibers [4],[7],[8]. For the tests for associations of the local ancestry, we additionally adjusted for other cardiovascular risk factors.

For the purpose of this dichotomous admixture analysis, study participants were ranked by the adjusted values for each trait. For CRAE, the 15% of individuals with the lowest values were defined as cases, and the 15% with the highest values as controls. For CRVE, conversely, the 15% with the highest values for were defined as cases, and the 15% with the lowest values as controls. Because ANCESTRYMAP uses Bayesian statistics, a prior distribution of risk models is required [23]. We tested 18 pre-specified European ancestry risk models to assess overall evidence of association by averaging across all models. The first 6 models used 0.4, 0.5, 0.67, 1.5, 2.0 and 2.5-fold risks of being a case due to inheritance of one copy of an European ancestral allele, with a risk of 1 for carrying zero copies of an European ancestral allele. The next 6 models used the same risk set as the first for carrying one copy of an European ancestral allele, whereas the risk of carrying zero copies were set to the reciprocal of the risks for carrying one copy. The last 6 models specified that inheritance of either one or two copies of an European ancestral allele had a risk of 1, but carrying zero copies had risks of 0.4, 0.5, 0.67, 1.5, 2.0 and 2.5. By convention used in the manuscript, a risk <1.0 for inheritance of one copy of an European ancestral allele at a given locus represents a risk model where European ancestry decreases risk relative to African ancestry. This set of models reflects the hypothesis that European ancestral alleles are less likely to confer risks but also tests for the alternative possibilities [23].

ANCESTRYMAP provided two scores to assess statistical significance: a *locus-specific score* and a *case-control score* [23]. A *locus-specific score* was obtained in cases (case-only analysis) by calculating the likelihood of the genotyping data at the SNPs at the locus under the risk model and comparing it to the likelihood of the genotyping data at the SNPs at the locus assuming that the locus is unassociated with the phenotype. The ratio of these two likelihoods is the “likelihood ratio”, and the log-base-10 of this quantity is the “LOD” score. A locus-specific LOD score of >5 has been recommended as criterion for genome-wide significance [37]. To obtain an assessment of the evidence for a risk locus anywhere in the genome, we averaged the likelihood ratio for association across all loci in the genome, and took the log10 to obtain a “genome-wide score”. We interpreted a genome-wide score >2 as significant [37].

A *case-control score* was calculated by comparing locus-specific deviations in European ancestry in cases versus controls at each locus across the genome. This score tests whether any deviation in ancestry from the genome-wide average is significantly different comparing cases with controls [23]. If there is no locus associated with phenotype, the case-control score is expected to be distributed approximately according to a standard normal distribution. For loci identified by the case-control score, the level of genome-wide significance was defined as a Z score >4.06 or <−4.06, corresponding to an uncorrected nominal *P*<5×10^−5^, or a corrected nominal *P*<0.05 after conservatively correcting for 1,000 hypotheses tested (approximately equals the number of independent chromosomal chunks assigned to either African or European ancestry).

### Assessing association of the retinal venular caliber to local ancestry at admixture-generated signals

The main admixture scans were based on a dichotomous phenotype (i.e., cases and controls) in a subset of our samples. To check whether the results were consistent in our entire sample, we used the ANCESTRYMAP software to obtain local estimates of African ancestry at the admixture peak [23], and then assessed the association of the local ancestry with retinal venular caliber as a continuous trait using linear regression models. Using ANCESTRYMAP, we also obtained regional estimates of ancestry based on the SNPs on chromosome 6p in the admixture panel. In addition to the five covariates used in the main admixture scans, CRVE was further adjusted for HDL cholesterol, LDL cholesterol and plasma triglyceride level, BMI, smoking, and alcohol consumption, all of which covariates have been shown to affect retinal vascular calibers [6]–[8],[14]. We then performed a normal-quantile transformation for CRVE to ensure normality. In the linear regression models, the transformed CRVE was used as a dependent variable and the local estimates of ancestry as an independent variable. To determine whether there was evidence of residual association with local ancestry after adjustment for global and regional ancestry, we included each individual's PAA and estimated regional ancestry on chromosome 6p as covariates in the regression models. This enabled us to increase power by including all samples in a quantitative analysis, rather than using only a subset of samples with the highest 15% and lowest 15% values in the dichotomous admixture scans described above. To determine whether the association between the local ancestry and CRVE differed significantly by hypertension status, Z tests were used to compare the difference in the regression coefficients obtained from the hypertensive and non-hypertensive groups.

### Credible interval for the position of a genetic locus

To determine a 95% CI for the position of a trait locus, we obtained the likelihood ratio for association at each marker on the chromosome where we identified an association. This provided a Bayesian posterior probability for the position of the underlying causal variant assuming a flat prior distribution across the region for the position of the trait locus. We defined the CI as the central region of this peak containing 95% of the area.

## Supporting Information

Figure S1 Genome-wide admixture mapping scans for loci affecting retinal arteriolar caliber in hypertensive individuals.
(0.19 MB PDF)

Figure S2 Posterior probability distribution for the position of the locus of retinal venular caliber.
(0.37 MB PDF)

Table S1 Summary of the initial admixture scan results by chromosome.
(0.06 MB DOC)

Table S2 Admixture scan results and association of the CRVE to local African ancestry at regions near 6p21.1 in the hypertensive African Americans.
(0.05 MB DOC)
