# Supplementary material for: Admixture Mapping Scans Identify a Locus Affecting Retinal Vascular Caliber in Hypertensive African Americans: the Atherosclerosis Risk in Communities (ARIC) Study
Source: PLoS Genet. 2010 Apr 15;6(4):e1000908. doi: 10.1371/journal.pgen.1000908 (PMC2855324; doi:10.1371/journal.pgen.1000908)

Figure S1. Genome-wide admixture mapping scans for loci affecting retinal arteriolar caliber in hypertensive individuals. For case-only analysis (LOD score, red line), the genome-wide score was 0.18, which did not reaching the threshold of >2 for significance. The highest locus-specific LOD was 2.37, arising from chromosome 5, followed by a LOD score of 2.04 on chromosome 6. For case-control analysis (Z score, blur gray line), the highest Z score was 3.73 (P = 1.91 x 10^{-4}), located on chromosome 6.

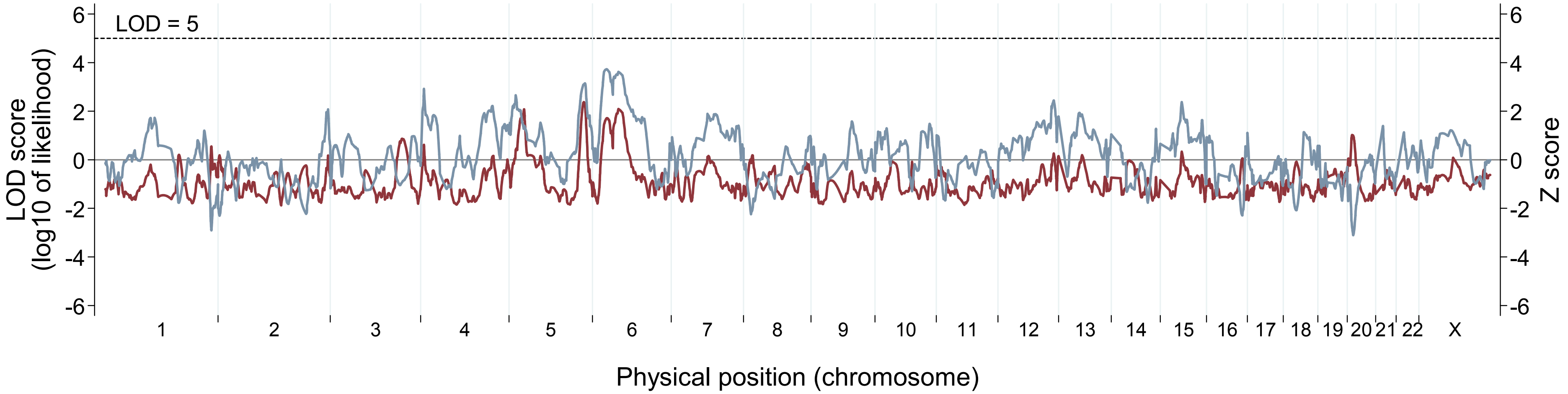

Supplement: Figure S1 — Genome-wide admixture mapping scans for loci affecting retinal arteriolar caliber in hypertensive individuals. (0.19 MB PDF) [file pgen.1000908.s001.pdf]
