# Supplementary material for: Admixture Mapping Scans Identify a Locus Affecting Retinal Vascular Caliber in Hypertensive African Americans: the Atherosclerosis Risk in Communities (ARIC) Study
Source: PLoS Genet. 2010 Apr 15;6(4):e1000908. doi: 10.1371/journal.pgen.1000908 (PMC2855324; doi:10.1371/journal.pgen.1000908)
Supplement: Table S1 — Summary of the initial admixture scan results by chromosome. (0.06 MB DOC) [file pgen.1000908.s003.doc]

**Table S1. Summary of the initial admixture scan results by chromosome**

| **Chr.** | **CRAE** | | |  | **CRVE** | | |
| --- | --- | --- | --- | --- | --- | --- | --- |
|  | **Highest locus-specific LOD score** | **Highest case-control Z score** | **Lowest case-control Z score** |  | **Highest locus-specific LOD score** | **Highest case-control Z score** | **Lowest case-control Z score** |
| 1 | 0.49 | 2.71 | -3.03 |  | -0.22 | 2.19 | -2.67 |
| 2 | 0.26 | 1.67 | -2.50 |  | 0.32 | 2.30 | -1.16 |
| 3 | 1.35 | 0.69 | -2.01 |  | -0.04 | 1.41 | -2.51 |
| 4 | 0.93 | 3.03 | -1.36 |  | -0.07 | 1.52 | -1.89 |
| 5 | 0.52 | 2.17 | -0.92 |  | 0.60 | 0.59 | -2.21 |
| 6 | 0.41 | 2.71 | -1.23 |  | 1.45 | 0.87 | -3.06 |
| 7 | -0.56 | 1.58 | -0.83 |  | -0.53 | 0.87 | -1.75 |
| 8 | -0.07 | 1.93 | -1.46 |  | -0.30 | 0.83 | -2.28 |
| 9 | -0.78 | 2.02 | -0.94 |  | -0.02 | 2.42 | -1.45 |
| 10 | -0.41 | 1.51 | -2.01 |  | -0.28 | 2.11 | -1.11 |
| 11 | -0.36 | 1.24 | -2.32 |  | 0.01 | 1.43 | -0.61 |
| 12 | -0.34 | 1.95 | -0.54 |  | 0.36 | 0.42 | -2.47 |
| 13 | -0.86 | 1.30 | -0.50 |  | 0.70 | 0.33 | -1.80 |
| 14 | -0.28 | 1.15 | -0.90 |  | -0.48 | 2.13 | -1.26 |
| 15 | 0.23 | 2.85 | -0.24 |  | -0.16 | 0.41 | -1.49 |
| 16 | 0.55 | 1.99 | -2.71 |  | -0.05 | 1.10 | -2.01 |
| 17 | -0.94 | 0.86 | -1.46 |  | -0.98 | 1.10 | -1.54 |
| 18 | -0.57 | 0.78 | -0.83 |  | 0.48 | 2.65 | -1.26 |
| 19 | -0.38 | 0.65 | -2.19 |  | -1.52 | 1.00 | -1.40 |
| 20 | -1.08 | 0.63 | -1.31 |  | -0.70 | 1.51 | -1.11 |
| 21 | -0.84 | 1.22 | -1.80 |  | -0.78 | 0.81 | -0.98 |
| 22 | -0.73 | 1.75 | -0.22 |  | 0.59 | 0.04 | -2.56 |
| X | 0.28 | 0.96 | -1.46 |  | 0.17 | 2.59 | -1.80 |

CRAE, central retinal artery equivalent; CRVE, central retinal vein equivalent.
