# Supplementary material for: Admixture Mapping Scans Identify a Locus Affecting Retinal Vascular Caliber in Hypertensive African Americans: the Atherosclerosis Risk in Communities (ARIC) Study
Source: PLoS Genet. 2010 Apr 15;6(4):e1000908. doi: 10.1371/journal.pgen.1000908 (PMC2855324; doi:10.1371/journal.pgen.1000908)
Supplement: Table S2 — Admixture scan results and association of the CRVE to local African ancestry at regions near 6p21.1 in the hypertensive African Americans. (0.05 MB DOC) [file pgen.1000908.s004.doc]

**Table S2.** Admixture scan results and association of the CRVE to local African ancestry at regions near 6p21.1 in the hypertensive African Americans

|  |  |  | **Linear regression analysis^a^** | | | | |
| --- | --- | --- | --- | --- | --- | --- | --- |
| **SNP** | **Physical position on chromosome 6 (Mb)** | **Locus-specific LOD from ANCESTRYMAP** | **Reg. Coef.^b^** | **(95% CI)** | ***P* value** | ***P* value, global** | ***P* value, global and regional** |
| rs11966463 | 22.11 | -0.32 | 0.18 | (0.04, 0.31) | 0.013 | 0.128 | 0.819 |
| rs303016 | 25.25 | 0.24 | 0.21 | (0.07, 0.34) | 0.003 | 0.039 | 0.301 |
| rs11962335 | 35.55 | 0.90 | 0.20 | (0.07, 0.33) | 0.003 | 0.032 | 0.240 |
| rs2181243 | 39.33 | 2.56 | 0.29 | (0.16, 0.43) | 2.7 x 10^-5^ | 0.001 | 0.005 |
| rs619572 | 42.43 | 5.51 | 0.36 | (0.23, 0.48) | 2.6 x 10^-8^ | 7.1 x 10^-7^ | 3.8 x 10^-6^ |
| rs1334601 | 43.76 | 5.04 | 0.34 | (0.22, 0.46) | 7.1 x 10^-8^ | 1.7 x 10^-6^ | 1.0 x 10^-5^ |
| rs7757763 | 44.09 | 4.06 | 0.32 | (0.19, 0.44) | 4.5 x 10^-7^ | 1.0 x 10^-5^ | 7.4 x 10^-5^ |
| rs927485 | 44.65 | 3.63 | 0.32 | (0.19, 0.44) | 4.7 x 10^-7^ | 1.1 x 10^-5^ | 8.0 x 10^-5^ |
| rs10948308 | 46.86 | 3.00 | 0.31 | (0.19, 0.44) | 7.5 x 10^-7^ | 1.9 x 10^-5^ | 1.3 x 10^-4^ |
| rs243123 | 48.74 | 2.77 | 0.30 | (0.18, 0.42) | 1.7 x 10^-6^ | 4.3 x 10^-5^ | 3.3 x 10^-4^ |
| rs7766458 | 54.25 | 2.94 | 0.29 | (0.17, 0.41) | 2.3 x 10^-6^ | 5.8 x 10^-5^ | 4.7 x 10^-4^ |
| rs9475487 | 55.93 | 2.95 | 0.29 | (0.17, 0.41) | 2.3 x 10^-6^ | 5.8 x 10^-5^ | 4.5 x 10^-4^ |

CRVE, central retinal vein equivalent; Reg. Coef., regression coefficient of the local ancestry; CI, confidence interval.

CRVE was adjusted for age, sex, study site, 6-year mean arterial pressure, and fasting glucose level.

SNPs in the 95% credible interval for the 6p21.1 locus are in the shaded area.

^a^ CRVE was normal-quantile transformed in the linear regression analysis. Regression coefficient and *P* values are unadjusted. *P* values adjusted for global ancestry and *P* values adjusted for both global and regional ancestry are also presented.

^b^ Regression coefficient of the local ancestry represents the change in Z score for each additional copy of the local ancestry allele.
