# Supplementary material for: Admixture Mapping Scans Identify a Locus Affecting Retinal Vascular Caliber in Hypertensive African Americans: the Atherosclerosis Risk in Communities (ARIC) Study
Source: PLoS Genet. 2010 Apr 15;6(4):e1000908. doi: 10.1371/journal.pgen.1000908 (PMC2855324; doi:10.1371/journal.pgen.1000908)

Figure S2. Posterior probability distribution for the position of the locus of retinal venular caliber. The 95% credible interval spanned from 40.840992 to 43.916949 Mb (marked by gray dash lines) on build 35 of the human genome reference sequence and contained many genes (diagram was taken from the UCSC Genome Browser: http://genome.ucsc.edu/cgi-bin/hgTracks?hgS_doOtherUser=submit&hgS_otherUserName=Cycheng&hgS_otherUserSessionName=95CIof6p21).

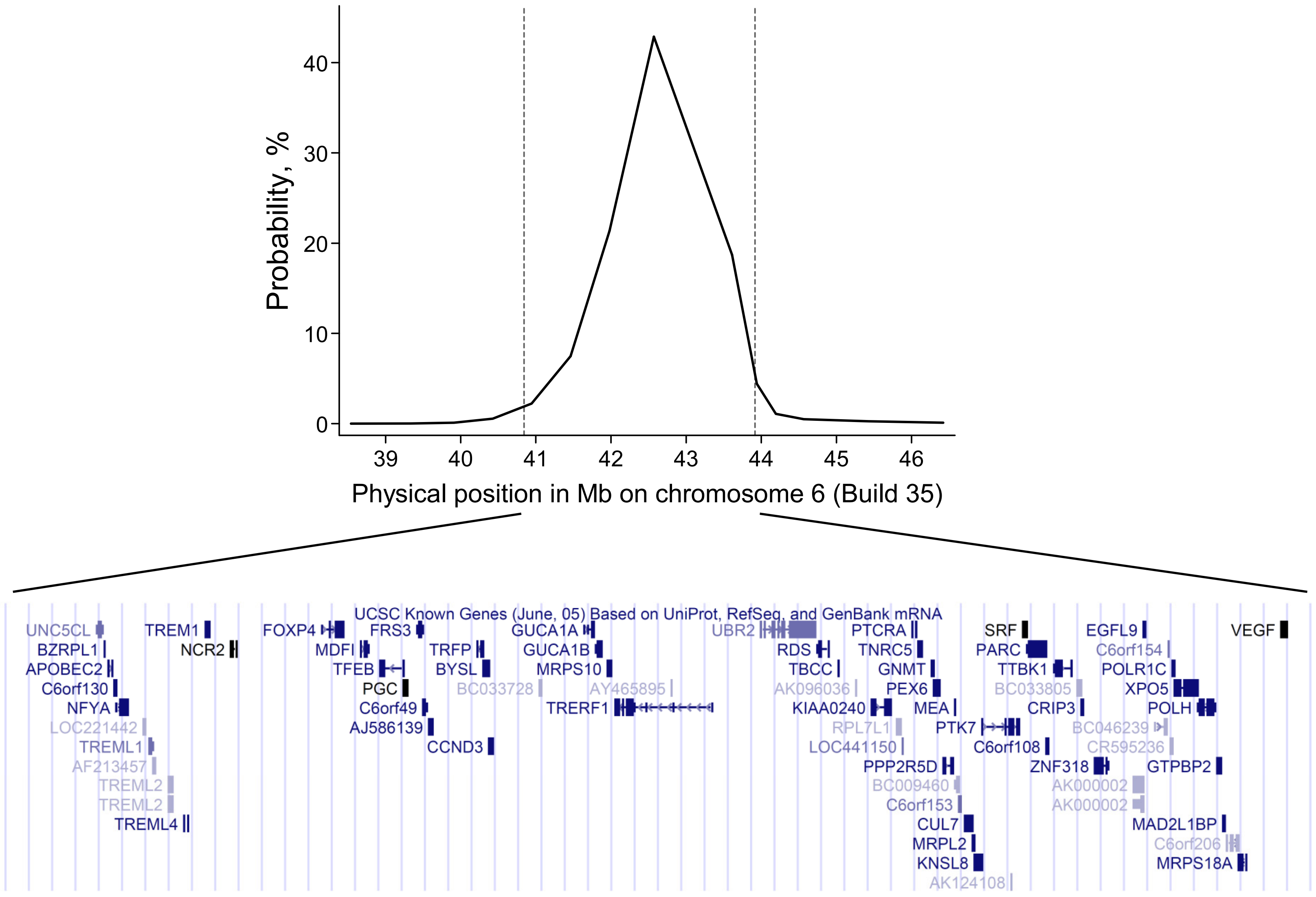

Supplement: Figure S2 — Posterior probability distribution for the position of the locus of retinal venular caliber. (0.37 MB PDF) [file pgen.1000908.s002.pdf]
